# Translations

**Published:** 1881-10

**Authors:** William D. Kempton


### TRANSLATIONS.

BY WILLIAM D. KEMPTON, M.D., D.D.S.

Treatment of deformities of the jaw and displacement of the teeth, due to the extracting of one or more, by means of artificial dentures.

The deformity of the jaws and displacement of the teeth can be prevented only by a timely adaptation of a denture, by this means any change in the expression or in the articulation is avoided, and the dentist has little trouble in supplying the deficiency, whilst in cases of long standing, the change in the position of the teeth renders it difficult unless one wants to extract all that remain, a proceedure that is not always advisable. Healthy roots should be smoothed off and filled, as the absorption of the alveolus following their extraction would necessitate too great a thickness of plate. It is often difficult when taking the articulation for full lower sets to get the jaw to close as far back as it should, and even when we do, we sometimes find that it protrudes beyond the upper one. This elongation of the jaw occurs in cases where the molars have been lost first. In such cases the teeth should be set high up, and if no incisor roots are present, they can be set far enough back to make an æsthetic denture, but if from the presence of roots they cannot be set far enough back to strike inside of the upper ones, they ought at least to strike on their cutting edges.

In adapting dentures where the remaining teeth are much displaced, various methods are advisable. The molars should be supplied by all means, as it is only by the most complete restoration that the patient will be benefitted. A very small space may be enlarged by a corundum disk, so as to admit a bicuspid or a small molar, or, if too small, it should be filled with rubber high enough to articulate with the antagonizing tooth.

When the lower incisors strike the gum in the upper jaw, the molars can be set down a little lower or a small piece of gold plate can be inserted, if the base is rubber, for the incisors to strike against. (*G. Juterbock in Vierteljahresschrift des Vereins Deutcher Zahn-Kunstler for August*, 1881.)

Query by translator. Why not cut $\frac{1}{16}$ of an inch off the lower incisors with a stump corundum wheel?

